# Characterization of BrGH3A, a bovine rumen-derived glycoside hydrolase family 3 *β*-glucosidase with a permuted domain arrangement

**DOI:** 10.1371/journal.pone.0305817

**Published:** 2024-07-09

**Authors:** Phiraya Pitchayatanakorn, Eukote Suwan, Prachumporn T. Kongsaeree

**Affiliations:** 1 Department of Biochemistry, Faculty of Science, Kasetsart University, Bangkok, Thailand; 2 Department of Veterinary Technology, Faculty of Veterinary Technology, Kasetsart University, Bangkok, Thailand; INRAE, FRANCE

## Abstract

The bovine rumen contains a large consortium of residential microbes that release a variety of digestive enzymes for feed degradation. However, the utilization of these microbial enzymes is still limited because these rumen microorganisms are mostly anaerobes and are thus unculturable. Therefore, we applied a sequence-based metagenomic approach to identify a novel 2,445-bp glycoside hydrolase family 3 *β*-glucosidase gene known as *BrGH3A* from the metagenome of bovine ruminal fluid. BrGH3A *β*-glucosidase is a 92-kDa polypeptide composed of 814 amino acid residues. Unlike most glycoside hydrolases in the same family, BrGH3A exhibited a permuted domain arrangement consisting of an (*α*/*β*)_6_ sandwich domain, a fibronectin type III domain and a (*β*/*α*)_8_ barrel domain. BrGH3A exhibited greater catalytic efficiency toward laminaribiose than cellobiose. We proposed that BrGH3A is an exo-acting *β*-glucosidase from *Spirochaetales* bacteria that is possibly involved in the intracellular degradation of *β*-1,3-/1,4-mixed linkage glucans that are present in grass cell walls. BrGH3A exhibits rich diversity in rumen hydrolytic enzymes and may represent a member of a new clan with a permuted domain topology within the large family.

## Introduction

Glycoside hydrolases are a group of hydrolases that act on glycosidic bonds with highly diverse specificities. Glucoside hydrolases are classified into families based on their structural similarities, as curated by the CAZy database (http://www.cazy.org) [1]. The glycoside hydrolase (GH) family 3 is a group of exo-acting, structure-related carbohydrate-active enzymes that exhibit a variety of activities, including *β*-D-glucosidases, *α*-L-arabinosidases, *β*-D-xylosidases, N-acetyl-*β*-D-glucosaminidases, and N-acetyl-*β*-D-glucosaminide phosphorylases [2]. GH3 members are retaining enzymes that act via a double-displacement mechanism involving the formation of two oxocarbenium ion-like transition states [3]. GH3 enzymes play many vital physiological roles, such as biomass conversion, cell wall and energy metabolism, and host-pathogen interactions; in particular, their ability to deconstruct plant polysaccharides has attracted considerable interest due to their potential application in biorefinery processes [4, 5].

Ruminant animals possess four stomach compartments: the reticulum, rumen, omasum, and abomasum. In the rumen, the digested feeds (such as straw, hay, and grass) are degraded into glucose and other constituent monosaccharides, which are subsequently converted into volatile fatty acids; all of these activities are accomplished by the activities of enzymes produced by the residential microbiota. Thus, there is a strong interest in the microbial ecosystems in the rumen, which include bacteria, fungi, protozoa, archaea, and bacteriophages, as well as the utilization of their enzymes [6]. However, these microbes are anaerobes and are thereby unculturable via the standard methods. To overcome this problem, the metagenomic approach has been applied to investigate the genomes that are directly acquired from environmental samples without culturing individual organisms [7]. Metagenomic studies of the rumen microflora in various animals have demonstrated the presence of a variety of carbohydrate-degrading enzymes [8]. In particular, several genes encoding GH3 *β*-glucosidases, such as Rubg3A and Rubg3B from yak (*Bos grunniens*) [9], Unglu135B12 from cattle feeding with *Miscanthus sinensis* [10], SRF2g14, SRF2g18, LAB20g4 and LAB25g2 from bovine ruminal fiber [4], and GlyA_1_ from bovine ruminal fluid [11], have been identified from rumen metagenomes. In addition, two GH1 *β*-glucosidases from bovine and sheep rumen metagenomes have been characterized [12–14].

In this study, we used a metagenomic approach to identify *BrGH3A*, which is a novel GH3 *β*-glucosidase gene, from bovine ruminal fluid. Although the typical three-domain GH3 enzymes contain an N-terminal (*β*/*α*)_8_ barrel-like domain, followed by an (*α*/*β*)_6_ sandwich domain and a fibronectin type III (FnIII) domain at the C-terminus [15], our BrGH3A exhibited a permuted domain arrangement with the (*β*/*α*)_8_ domain located at the C-terminus. We performed the complete enzymatic characterization of BrGH3A, including its specificity toward various natural disaccharide substrates. Its apparent high catalytic efficiency toward laminaribiose suggested its role in the breakdown of *β*-1,3-glucans that are present in grass cell walls. Our results highlight the diversity of hydrolytic enzymes that are present in the rumen microbial community and the potential of the metagenomic approach in the discovery of unique enzymes from this rich resource.

## Materials and methods

### Extraction of metagenomic DNA

A total of six samples, which included the ruminal content, fluid, and endothelium of two cows, were collected at the slaughterhouse in Rangsit, Pathum Thani, Thailand, and maintained on ice in airtight containers. Metagenomic DNA was extracted from the rumen samples within 6 h after sample collection by using the QIAamp® DNA Stool Mini Kit (Qiagen, Germany). The quantity and quality of the extracted metagenomic DNA were analyzed by using a NanoDrop spectrophotometer (Thermo Scientific, USA) and 1% agarose gel electrophoresis.

### *In silico* analysis and design of degenerate primers

The existing sequences of *β*-glucosidases in rumen microorganisms were retrieved from the National Center for Biotechnology Information (NCBI) database [16] by using the terms “glucosidase” or “glucoside hydrolase” from rumen microorganism genomic data. The GH families were classified by using the conserved domain database [17]. The full-length amino acid sequences of enzymes in GH3 were aligned, and phylogenetic trees were constructed by using multiple sequence alignment [18]. Two pairs of degenerate primers (NCF_for and NCF_rev; CFN_for and CFN_rev) were designed based on conserved nucleotide sequences of the N- and C-terminal domains of GH3 *β*-glucosidases (S1 Table).

### Amplification of partial fragments of GH3 *β*-glucosidase genes

A total of twelve PCRs were performed by using two pairs of degenerate primers to screen for partial sequences of GH3 *β*-glucosidase in the 6 metagenomic DNA samples. The quantity and quality of the PCR products were analyzed by using 1% agarose gel electrophoresis. The PCR products were then ligated into the pGEM®-T Easy Vector (Promega, Madison, WI, USA). *Escherichia coli* DH5α (Thermo Fisher Scientific, Waltham, MA, USA) was transformed with the ligation reactions and selected on LB agar supplemented with 100 μg/mL ampicillin, 0.5 mM isopropyl *β*-D-1-thiogalactopyranoside (IPTG), and 80 μg/mL X-gal at 37°C overnight. The nucleotide sequences of the inserted DNA fragments were determined via Sanger sequencing, analyzed by using BLAST [19] to identify regions of local similarity, and translated into amino acid sequences by using the Translate tool on the ExPASy server [20].

### Genome walking

The nucleotide sequences from the initial amplification were used to design gene-specific primers for primary and secondary PCR (GSP1 and GSP2, respectively) at both the 5’ and 3’ ends (S1 Table). Genome walking was performed by using the Universal GenomeWalker™ 2.0 kit (Clontech Laboratories, Mountain View, CA, USA). The nucleotide sequences of the genome walking products were determined. The overlapping DNA sequences from the initial amplification and genome walking were assembled to generate a draft full-length sequence of a GH3 *β*-glucosidase.

### Cloning of the full-length *BrGH3A β*-glucosidase gene

The full-length sequence of bovine rumen-derived GH3 *β*-glucosidase (known as *BrGH3A*) was amplified from the rumen metagenomic DNA samples by using a pair of 5’ and 3’ gene-specific primers (BrGH3A_for and BrGH3A_rev) containing the *Nde*I and *Bam*HI sites, respectively (S1 Table). The PCR product was ligated into pET15b at the *Nde*I-*Bam*HI sites to generate the recombinant plasmid pET15b-BrGH3A. *E*. *coli* DH5α was transformed with pET15b-BrGH3A and selected on LB agar supplemented with 100 μg/mL ampicillin at 37°C overnight. The nucleotide sequence of pET15b-BrGH3A was confirmed. The theoretical parameters of BrGH3A, including the molecular weight and pI, the presence of a signal peptide, and the structural model, were predicted by using ProtParam, SignalP-6.0, and AlphaFold2, respectively [20–22].

### Expression and purification of BrGH3A

*E*. *coli* BL21-CodonPlus(DE3)-RIL (Agilent Technologies, Santa Clara, CA, USA) was transformed with the recombinant plasmid pET15b-BrGH3A and selected on LB agar supplemented with 100 μg/mL ampicillin and 25 μg/mL chloramphenicol. The expression of BrGH3A was induced with 0.1 mM IPTG at 18°C for 20 h. The enzyme was purified from the soluble cell lysate, as previously described [12]. Western blot analysis was performed with HisProbe-HRP antibody and SuperSignal West Pico Chemiluminescent Substrate (Thermo Scientific, Rockford, IL, USA). The protein concentration was determined by using a Protein Assay Reagent Kit (Bio-Rad, Hercules, California, USA) and compared to a standard curve of bovine serum albumin.

### Effects of temperature and pH on enzyme activity

The optimal temperature for enzyme activity was determined by reacting BrGH3A with 15 mM *p*-nitrophenyl-*β*-D-glucopyranoside (*p*NP-Glc) in 0.1 M sodium citrate, pH 5.0, at 0–100°C for 5 min. The blanks contained 15 mM *p*NP-Glc in 0.1 M sodium citrate, pH 5.0, without the enzyme and were incubated at 0–100°C for 5 min.

The optimal pH for enzyme activity was determined by reacting BrGH3A with 15 mM *p*NP-Glc in 0.1 M sodium citrate, pH 2.0–6.5, and 0.1 M McIlvaine buffer, pH 3.0–9.0, at 40°C for 5 min. The blanks contained 15 mM *p*NP-Glc in the corresponding buffers without the enzyme and were incubated at 40°C for 5 min.

The temperature stability was determined by preincubating BrGH3A in 0.1 M sodium citrate, pH 5.0, at 0–100°C for 30 min before measuring the enzyme activity with 15 mM *p*NP-Glc in 0.1 M sodium citrate, pH 5.0, at 40°C for 5 min. The pH stability was determined by preincubating BrGH3A in 0.1 M sodium citrate, pH 2.0–6.0, and 0.1 M McIlvaine buffer, pH 4.0–9.0, at 4°C for 30 min before measuring the enzyme activity with 15 mM *p*NP-Glc in 0.1 M sodium citrate, pH 5.0, at 40°C for 5 min. The blanks contained 15 mM *p*NP-Glc in 0.1 M sodium citrate, pH 5.0, without the enzyme and were incubated at 40°C for 5 min.

All of the reactions and blanks were stopped by adding 2 M sodium carbonate, and the absorbance was read at 400 nm. To account for the nonspecific hydrolysis of the substrate, the absorbance of the blank was subtracted from that of the corresponding reaction. The relative hydrolytic activity was expressed as a percentage of the corrected absorbance compared to that of the optimal assay condition. The data are reported as the means and standard errors from triplicate experiments.

### Hydrolysis of glucooligosaccharides

The purified BrGH3A was reacted with 1 mM glucooligosaccharides in 0.1 M sodium citrate, pH 5.0, at 40°C for 5 min. The reactions were stopped by boiling for 5 min. The released glucose was reacted with 2 mg/mL 2,2’-azino-bis(3-ethylbenzothiazoline-6-sulfonic acid) (ABTS) and glucose oxidase reagent at 37°C for 15 min, and the absorbance was read at 400 nm. The blanks contained 1 mM glucooligosaccharides in 0.1 M sodium citrate, pH 5.0, without the enzyme and were treated similarly. To account for the nonspecific hydrolysis of the substrates, the absorbance of each blank was subtracted from that of the corresponding reaction. The amount of glucose product was determined by comparing the corrected absorbance with a standard curve. Afterward, the values for disaccharides were determined by dividing the amount of released glucose by two because two glucose molecules were released from one substrate molecule in a single cleavage event. Hydrolysis of oligosaccharides may also yield more than one glucose molecule from one substrate molecule due to sequential cleavage; however, the values for oligosaccharides were calculated in terms of the total released glucose. The relative hydrolytic activity was expressed as a percentage of released glucose compared to the highest glucose yield. The data are reported as the means and standard errors from triplicate experiments.

To investigate the mode of enzymatic action, 2 μg of BrGH3A was incubated with 10 mM laminaritetraose in 20 mM sodium citrate, pH 5.0, at 40°C for 1 h, and aliquots (4 μL) were taken at different time intervals. The blanks contained 10 mM laminaritetraose in 0.1 M sodium citrate, pH 5.0, without the enzyme and were treated similarly. The reaction mixtures were separated by using thin layer chromatography (TLC) (Silica gel 60 F254; Merck, Darmstadt, Germany), which was developed twice in a mixture of 4:2:1 (v/v) 1-butanol:acetic acid:water. The spots were visualized by soaking the TLC plate in 20% sulfuric acid in ethanol and heating it until spots were observed.

To investigate the possible glucosyl transfer reactions, 20 μg of BrGH3A was incubated with 5–40 mM *p*NP-Glc in 20 mM sodium citrate, pH 5.0, at 40°C for 6 h, and aliquots (10 μL) were taken at different time intervals. The blanks contained 40 mM *p*NP-Glc in 20 mM sodium citrate, pH 5.0, without the enzyme and were treated similarly. The reaction products were separated by using TLC, which was developed twice in a mixture of 8:2:1 (v/v) 1-butanol:acetic acid:water. The spots were visualized as described above.

### Kinetic analysis

Kinetic parameters were determined by reacting the purified enzyme against various concentrations of *p*NP-Glc, *p*-nitrophenyl-*α*-L-arabinopyranoside (*p*NP-Ara), *p*-nitrophenyl-*β*-D-fucopyranoside (*p*NP-Fuc), *p*-nitrophenyl-*β*-D-galactopyranoside (*p*NP-Gal), *p*-nitrophenyl-*β*-D-xylopyranoside (*p*NP-Xyl), cellobiose, laminaribiose, laminaritriose, and xylobiose in 0.1 M sodium citrate, pH 5.0, at 40°C for 5 min. All of the reactions against *p*NP-glycosides were stopped by adding 2 M sodium carbonate, and the absorbance was read at 400 nm. All of the reactions against di- and trisaccharides were stopped by boiling for 5 min. The amount of glucose that was released from cellobiose, laminaribiose, and laminaritriose was determined as described above. The released xylose from xylobiose was reacted with 1.67% (w/v) para-bromoaniline reagent at 70°C for 10 min and then at 30°C for 60 min in the dark, and the absorbance was read at 520 nm [23]. The blanks contained the corresponding substrates in 0.1 M sodium citrate, pH 5.0, without the enzyme and were treated similarly. To account for the nonspecific hydrolysis of the substrates, the absorbance of the blank was subtracted from that of the corresponding reaction. The amount of product (*p*-nitrophenol, glucose, or xylose) was determined by comparing the corrected absorbance with a standard curve. The amounts of glucose (or xylose) that was released from disaccharides were divided by two because two glucose molecules were released from one substrate molecule in a single cleavage. Hydrolysis of laminaritriose may also yield more than one glucose molecule from one substrate molecule due to sequential cleavage; however, the values for laminaritriose were calculated in terms of the total released glucose. The data are reported as the means and standard errors from triplicate experiments.

Kinetic parameters (*K*_m_, *k*_cat,_ and *k*_cat_/*K*_m_) were calculated via nonlinear regression of the Michaelis–Menten equation by using the program KaleidaGraph (Synergy Software, Reading, PA, USA). In cases where the concentrations of the substrate were low compared with the *K*_m_ value, the Lineweaver‒Burk equation was used.

## Results and discussion

### Identification of a novel GH3 BrGH3A *β*-glucosidase with a permuted domain arrangement

The search results from the NCBI database for the *β*-glucosidase sequences from rumen microorganisms demonstrated 183 full-length GH3 sequences from 20 species. Most of these sequences belonged to the phylum Firmicutes, especially *Butyrivibrio fibrisolvens* and *Lachnospiraceae bacterium*. These sequences were aligned, and the relationships were analyzed via phylogenetic tree analysis [12]. Among these sequences, 132 contained three conserved domains: the GH3 N-terminal domain (pfam00933), the GH3 C-terminal domain (pfam01915), and the FnIII-like domain (pfam14310). These sequences were further divided into 2 groups based on their domain arrangements: a group of 68 sequences with an N-C-FnIII domain arrangement and a group of 58 sequences with a C-FnIII-N domain arrangement. Regardless of the domain arrangement, the N-terminal domains of these sequences contained the GR(N/T/L)(F/H/G)EY(Y/F)(S/P)ED(P/G) motif, whereas the C-terminal domains contained the PFG(F/Y/H)G(L/I)SYT motif. The nucleotide sequences of these conserved regions were used as a basis to design 2 pairs of forward and reverse degenerate primers, including NCF _for and NCF _rev, respectively, for the N-C-FnIII domain arrangement and CFN_for and CFN_rev, respectively, for the C-FnIII-N domain arrangement (S1 Table). These primers were used to amplify the partial gene fragments of GH3 *β*-glucosidases in six metagenomic DNA samples obtained from bovine rumens. The PCR products were then cloned and sequenced.

One of the clones harbored a 1,084-bp PCR product, which was a partial sequence of a GH3 *β*-glucosidase gene obtained from the amplification of the bovine ruminal fluid metagenome with the CFN_for and CFN_rev primers (S1 Fig). The deduced amino acid sequence shares the highest amino acid sequence identity (51%) with a *β*-glucosidase-related glycosidase from the uncultured bacterium scaffold00056 isolated from the cecum of the leaf-eating flying squirrel (*Petaurista alborufus* lena) (GenBank accession number AFN84577.1) and a GH3 N-terminal domain-containing protein from *Butyrivibrio* sp. AC2005 (GenBank accession number WP_044930885.1) [24, 25]) (S2 Fig). Therefore, the genome walking method was applied to obtain the complete coding sequence of this novel GH3 *β*-glucosidase (*BrGH3A*), which is 2,445 bp long (GenBank accession number ON683414). The deduced amino acid sequence of BrGH3A predicted a polypeptide of 814 residues, with an expected size of 91.7 kDa and a calculated pI of 6.6, but without a signal peptide (S3 Fig). The PFGFGLSYT motif of the C-terminal domain and the GRNFEYYSEDP motif of the N-terminal domain are located at positions 273–281 and 625–635 of BrGH3A, respectively. The amino acid sequence of BrGH3A shows 98% identity to the partial sequence of a GH3 C-terminal domain-containing protein from *Spirochaetales* bacterium from the goat rumen metagenome (GenBank accession number MBQ7644118.1, unpublished), 69% identity to a GH3 C-terminal domain-containing protein from the buffalo rumen metagenome (GenBank accession number MDT3388973.1, unpublished), and 67% identity to a GH3 C-terminal domain-containing protein from *Spirochaetales* bacterium from the camel rumen (GenBank accession number MCR5442458.1) [26], all of which have yet to be characterized.

**Fig 1:**
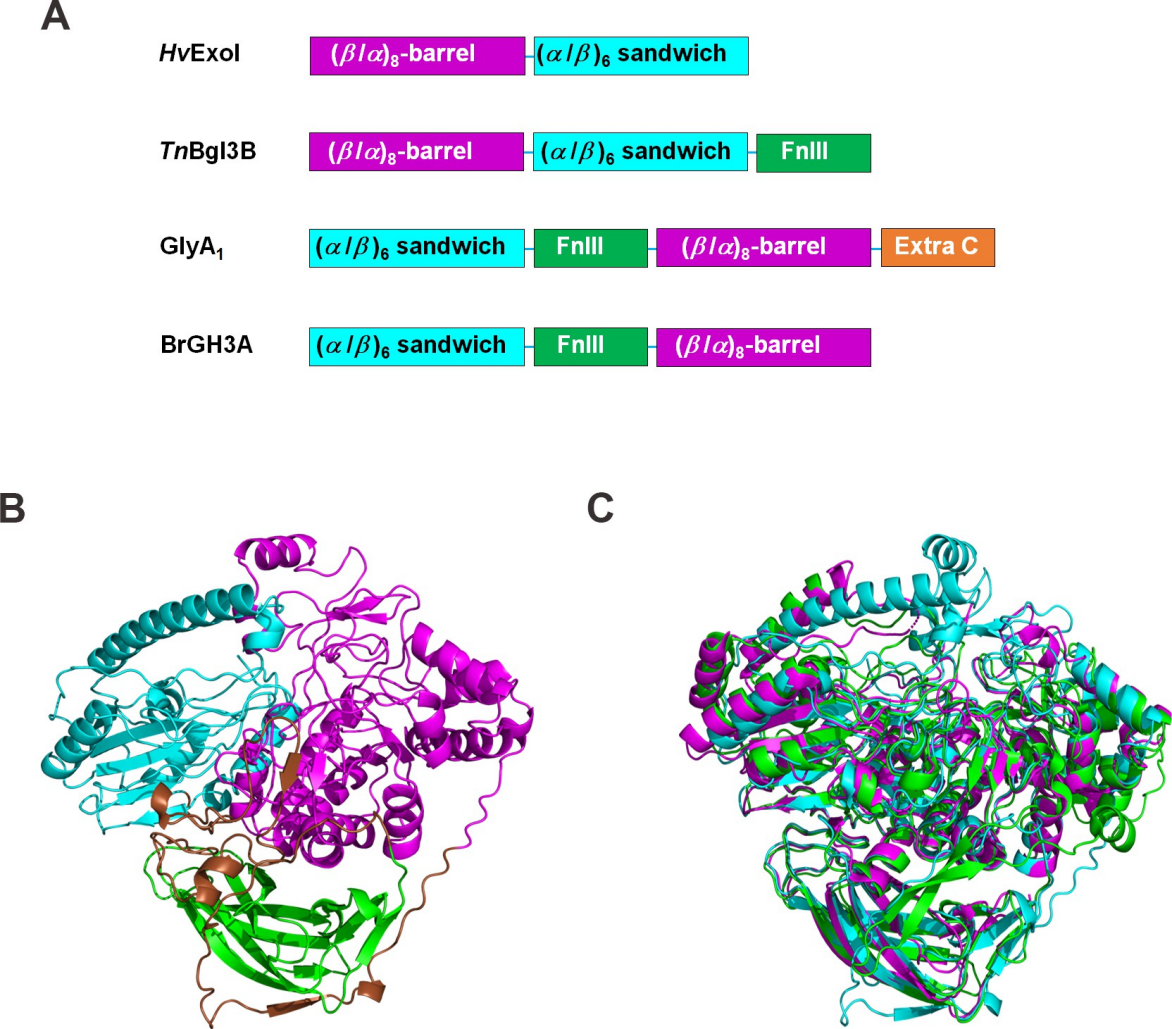
Domain arrangements and structures of representative GH3 enzymes. (A) Comparison of domain arrangements in *Hv*ExoI [25], *Tn*Bgl3B [28], GlyA_1_ [11], and BrGH3A. (B) The structural model of BrGH3A. The (*β*/*α*)_8_ barrel domains (magenta), the (*α*/*β*)_6_ sandwich domains (cyan), the FnIII domains (green), and the linker regions (brown) are indicated. The additional C-terminal domain in GlyA_1_ is shown in orange. (C) Superimposition of the structural model of BrGH3A (cyan) and the structures of *Tn*Bgl3B (PDB code 2X41, magenta) and GlyA_1_ (PDB code 5K6L, green).

**Fig 2:**
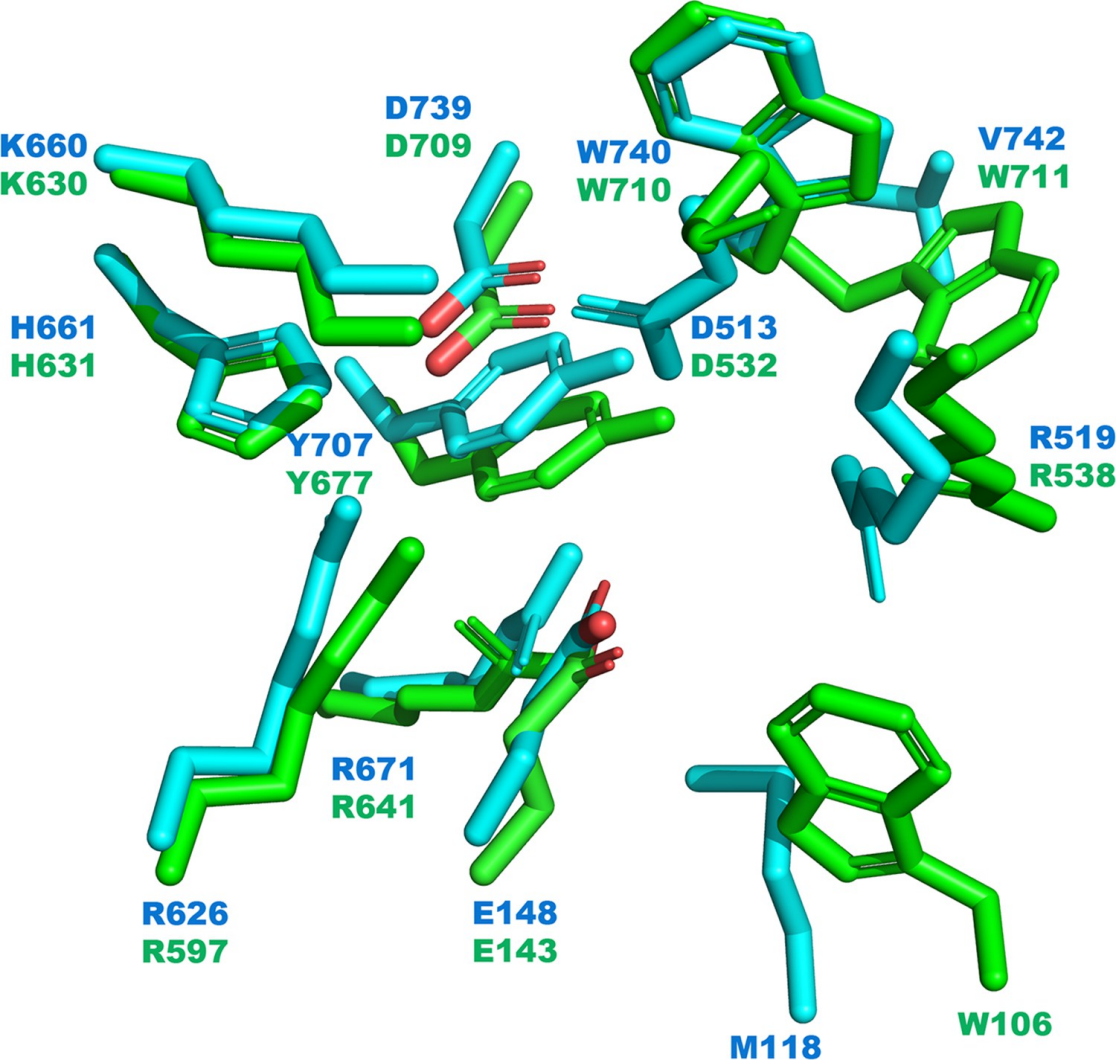
Comparison of the active site architectures of BrGH3A (cyan) and GlyA_1_ (PDB code 5K6L, green).

The structural model of BrGH3A, which was generated by AlphaFold2, is predicted to contain three domains that adopt the C-FnIII-N topology, which are the (*α*/*β*)_6_ sandwich domain (residues Met1-Arg221), the FnIII domain (residues Ser282-Pro406), and the (*β*/*α*)_8_ barrel-like domain (residues Asp448-Val814) (Fig 1A and 1B, and S4 Fig). The three domains are linked by two short coils (residues Glu222-Thr281 and residues Cys407-Tyr447). The domain arrangement in BrGH3A is similar to that in two previously reported GH3 *β*-glucosidases, including GlyA_1_ from the cow rumen metagenome (PDB code 5K6L, 32% identity to BrGH3A) and a GH3 *Paenibacillus barengoltzii β*-glucosidase (PDB code 5WUG, 33% identity to BrGH3A) [11, 27] (Fig 1A and S4 Fig). However, BrGH3A lacks the additional C-terminal domain that is present in both GlyA_1_ and *P*. *barengoltzii β*-glucosidase. By comparison, the seminal structure of the GH3 *β*-D-glucan exohydrolase from *Hordeum vulgare* (*Hv*ExoI) contains two domains, including the N-terminal (*β*/*α*)_8_ barrel domain and the C-terminal (*α*/*β*)_6_ sandwich domain (PDB code 1EX1), whereas GH3 *β*-glucosidase 3B from *Thermotoga neapolitana* (*Tn*Bgl3B) and many others possess the first two domains of *Hv*ExoI plus the third FnIII domain (PDB code 2X41) [25, 28] (Fig 1A). The structural model of BrGH3A superimposes well with the structures of both GlyA_1_ and *Tn*Bgl3B (Fig 1C). Based on sequence homology and structural alignment, the catalytic acid/base residue of BrGH3A is likely Glu148 located on the (*α*/*β*)_6_ sandwich domain, which corresponds to Glu143 of GlyA_1_ [11] and Glu458 of *Tn*Bgl3B [28]. Similarly, the nucleophile of BrGH3A is likely Asp739 on the (*β*/*α*)_8_ barrel domain, which corresponds to Asp709 of GlyA_1_ [11] and Asp 242 of *Tn*Bgl3B [28] (Fig 2 and S4 Fig).

A comparison of the active site structures demonstrated that the residues making up subsite –1 of GlyA_1_ and BrGH3A are well conserved, which is consistent with their affinity for glucoside substrates. For GlyA_1_, these residues are Glu143 (acid/base catalyst), Asp532, Arg597, Lys630, His631, Arg641, Tyr677, Asp709 (nucleophile), and Trp710 [11], which correspond to Glu148 (acid/base catalyst), Asp513, Arg626, Lys660, His661, Arg671, Tyr707, Asp739 (nucleophile), and Trp740 in BrGH3A, respectively (Fig 2 and S4 Fig). Conversely, the residues within subsite +1 are less conserved. For GlyA_1_, the putative residues are Trp106, Arg538, and Trp711, all of which are located in the flexible loop regions of the (*α*/*β*)_6_ sandwich domain and the (*β*/*α*)_8_ barrel domain. The corresponding residues in BrGH3A could be Met118, Arg519, and Val742 (Fig 2). Furthermore, the model suggested that BrGH3A possesses three loops (residues Thr472-Ala486, Ile521-Gln568, and Val741-Pro759) within the (*β*/*α*)_8_ barrel domain that may obstruct entry to the active site of BrGH3A (S4 Fig). The first and second loops of BrGH3A are not present in the structure of GlyA_1_, whereas the third loop corresponds to loop *β*7-*α*7 of GlyA_1_ (residues Trp711-Thr726). The differences in the amino acid residues and the loop structures contribute to the unique catalytic activity and substrate specificity of BrGH3A.

### Expression and purification of BrGH3A

**Fig 3:**
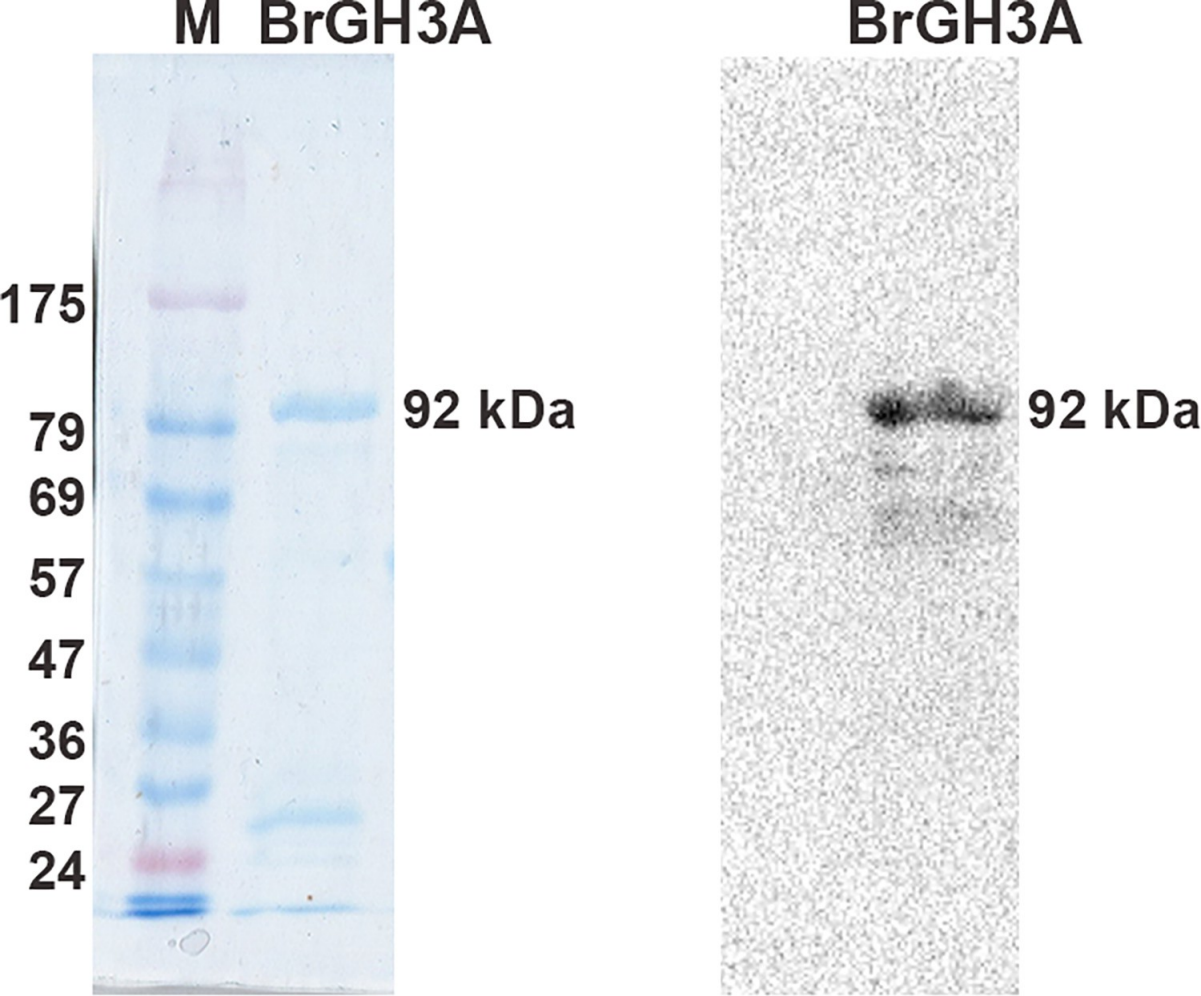
10% SDS‒PAGE analysis (left) and Western blot analysis (right) of the purified BrGH3A. Lane M is the protein size marker.

The coding sequence of BrGH3A was cloned, inserted into pET15b, and expressed from *E*. *coli* BL21-CodonPlus(DE3)-RIL with an N-terminal polyhistidine tag. BrGH3A was purified from a soluble cell lysate and appeared as a single-band protein of approximately 92 kDa, which could be detected by using an antibody against the polyhistidine tag (Fig 3).

### Effects of pH and temperature on enzyme activity

**Fig 4:**
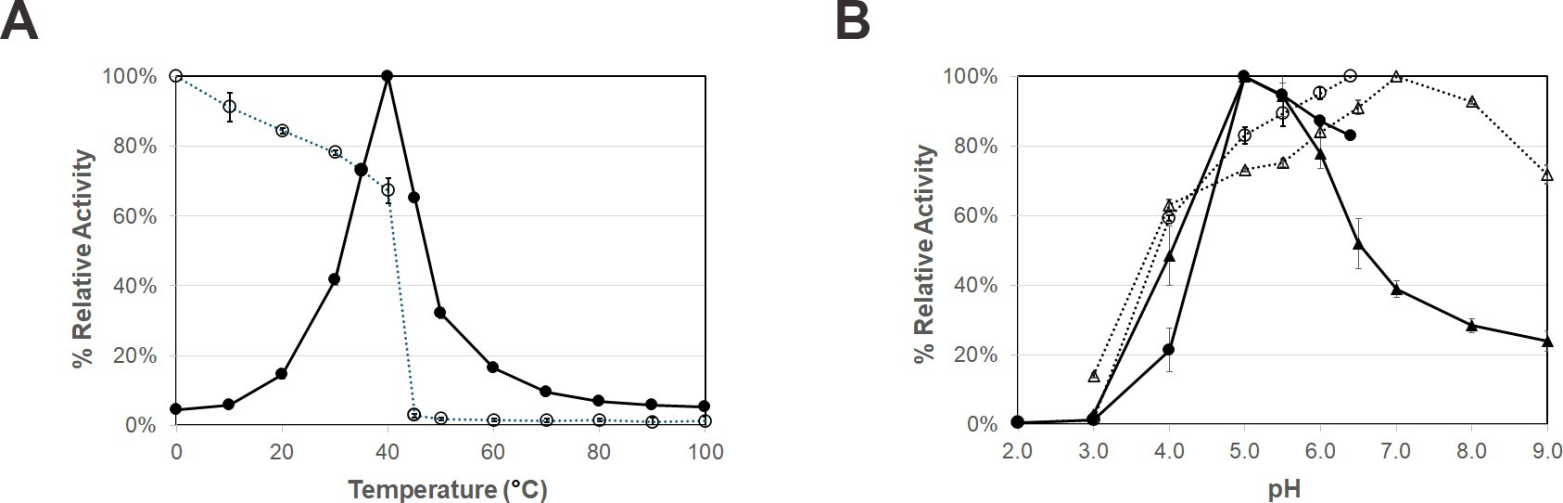
The effects of (A) temperature and (B) pH on the activity of BrGH3A. The optimal conditions are shown as filled symbols and solid lines, and the stability conditions are shown as open symbols and dotted lines. The effects of pH were tested in 0.1 M sodium citrate, pH 2.0–6.5 (circles), and 0.1 M McIlvaine buffer, pH 3.0–9.0 (triangles).

BrGH3A exhibited the optimal temperature and pH for the activity of BrGH3A at 40°C and pH 5.0, respectively (Fig 4A and 4B). BrGH3A exhibited at least 60% activity after preincubation at 0–40°C or pH 4.0–9.0 for 30 min. The enzyme lost activity when preincubated at over 45°C or below pH 3.0 for 30 min (Fig 4A and 4B). The optimal temperature and pH of BrGH3A agree well with the growth conditions of *Treponema saccharophilum*, which is a member of the order *Spirochaetales* that was previously isolated from bovine rumen fluid [29], and are similar to those of other GH3 *β*-glucosidases from ruminal metagenomes [4, 9, 10].

### Relative hydrolysis activity toward various substrates

The hydrolytic activity of BrGH3A toward glucooligosaccharides of various degrees of polymerization was expressed as a percentage relative to that toward laminaribiose, which provided the highest glucose yield (Table 1). After laminaribiose (*β*-1,3-glucosidic linkage), BrGH3A preferred cellobiose (*β*-1,4-glucosidic linkage) much more than gentiobiose (*β*-1,6-glucosidic linkage) and sophorose (*β*-1,2-glucosidic linkage). Both *β*-1,3- and *β*-1,4-linkages are present in plant cell wall polysaccharides in the form of cellulose and hemicellulose [8]. The hydrolysis of laminaritriose was also appreciably greater than that of cellotriose. In addition, BrGH3A showed a preference for di- and trisaccharides over longer-chain substrates, thus suggesting a short substrate binding pocket that may not accommodate long oligosaccharide chains.

**Table 1:** Relative hydrolytic activities of BrGH3A toward glucooligosaccharides.

Substrate	Relative activity^a^ (%)
Cellobiose^b^	17.4 ± 0.3
Cellotriose	13.1 ± 3.6
Cellotetraose	9.4 ± 2.5
Cellopentaose	4.3 ± 2.0
Laminaribiose^b^	100
Laminaritriose	20.1 ± 2.0
Laminaritetraose	4.0 ± 2.6
Laminaripentaose	2.0 ± 1.5
Sophorose^b^	0.9 ± 0.7
Gentiobiose^b^	3.0 ± 1.7

Note

^a^ Determined from the amount of released glucose compared to that from the hydrolysis of 1 mM laminaribiose.

^b^ The amounts of released glucose from disaccharides were divided by two because two glucose molecules were released from one substrate molecule in a single cleavage event.

**Fig 5:**
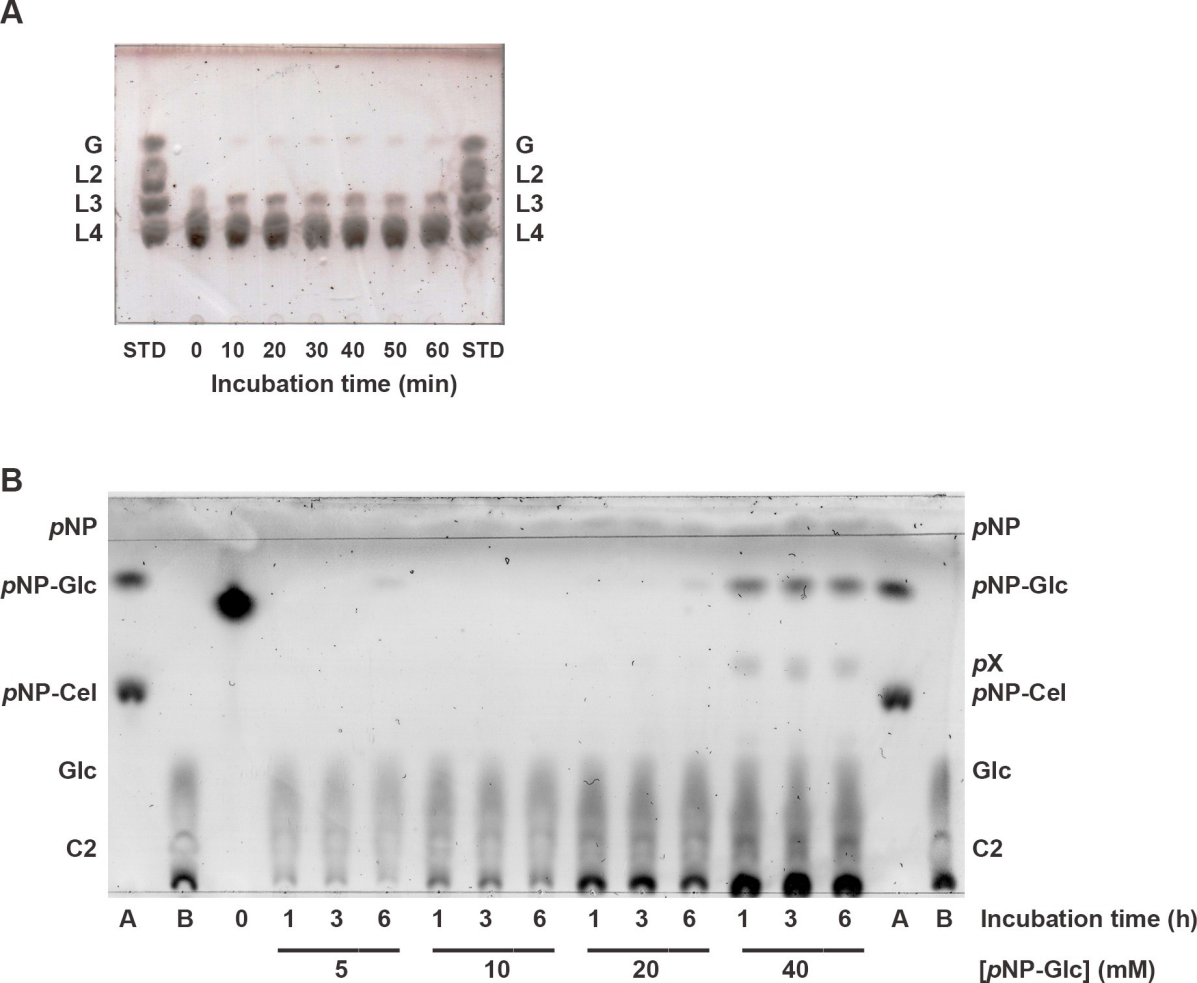
Time-course reactions of BrGH3A with laminaritetraose (A) and *p*NP-Glc (B). (A) The reaction consisted of 2 μg of BrGH3A and 10 mM laminaritetraose in 20 mM sodium citrate, pH 5.0, at 40°C for 1 h. The STD contained 20 nmol of each glucose (G), laminaribiose (L2), laminaritriose (L3), and laminaritetraose (L4). The incubation times are indicated below the lanes. (B) The reactions contained 20 μg of BrGH3A and 5–40 mM *p*NP-Glc in 20 mM sodium citrate, pH 5.0, at 40°C for 6 h. Lane A contains 60 nmol of *p*NP, 40 nmol of *p*NP-Glc, and 20 nmol of *p*NP-Cel. Lane B contains 40 nmol of each glucose (G) and cellobiose (C2). The incubation times and the concentrations of *p*NP-Glc are indicated below the lanes. The unknown product is labeled *p*X.

The time-course hydrolysis of laminaritetraose by BrGH3A yielded glucose and laminaritriose as initial hydrolysis products, thus indicating its exo-acting function (Fig 5A). The reactions of BrGH3A with 5–40 mM *p*NP-Glc over 6 h showed glucose and *p*NP as being the hydrolytic products, as well as some *p*NP-Glc (the remaining starting material) (Fig 5B). However, in the presence of a high *p*NP-Glc concentration (40 mM), an unknown product, which was likely a *p*NP-disaccharide, was observed between the *p*NP-Glc and *p*NP-*β*-D-cellobioside (*p*NP-Cel) bands. *p*NP-disaccharide could be formed by the transfer of the glucosyl moiety when the first *p*NP-Glc molecule acted as a glucosyl donor and the second *p*NP-Glc molecule acted as a glucosyl acceptor. Thus, BrGH3A possesses transglucosylation activity, which is common among GH3 *β*-glucosidases [30].

### Kinetic analysis

Kinetic analysis of BrGH3A toward various *p*NP-glycosides and oligosaccharides demonstrated that BrGH3A was highly specific toward the glucoside substrate with 56- to 17,000-fold greater efficiency (*k*_cat_/*K*_m_) toward *p*NP-Glc than other types of *p*NP-glycosides (Table 2). Minimal but appreciable catalytic activity was detected toward *p*NP-Xyl; however, no activity could be detected toward xylobiose. Among the two glucodisaccharides, the *K*_m_ of BrGH3A toward laminaribiose was 2-fold lower than that toward cellobiose; however, the *k*_cat_ was approximately 24-fold greater, thus resulting in an approximately 50-fold greater specificity for laminaribiose than for cellobiose. The hydrolysis of laminaritriose was approximately 3-fold less efficient than that of laminaribiose, thus suggesting a relatively short binding pocket with subsites +1 and +2 for di- and trisaccharide substrates, respectively. These kinetic data agree well with the relative hydrolytic activities (Table 1) and further confirm that BrGH3A is a *β*-glucosidase with a preference for *β*-1,3-linked substrates.

**Table 2:** Kinetic analysis of BrGH3A toward various *p*NP-glycosides and oligosaccharides.

Substrate	*K*_m_ (mM)	*k*_cat_ (s^-1^)	*k*_cat_ / *K*_m_ (M^-1^ s^-1^)
*p*NP-Glc	0.60 ± 0.04	119 ± 1	(200 ± 12) × 10^3^
*p*NP-Ara	9.95 ± 1.16	0.12 ± 0.01	12.0 ± 2.0
*p*NP-Fuc	7.96 ± 0.64	0.75 ± 0.06	93.8 ± 10.4
*p*NP-Gal	7.55 ± 0.90	0.54 ± 0.06	72.0 ± 11.9
*p*NP-Xyl	2.12 ± 0.10	7.45 ± 0.11	(3.52 ± 0.17) × 10^3^
Cellobiose	30.0 ± 3.9	0.47 ± 0.06	15.9 ± 2.9
Laminaribiose	14.3 ± 1.3	11.3 ± 0.9	791 ± 98
Laminaritriose	5.40 ± 0.42	1.42 ± 0.09	262 ± 26

Our BrGH3A enzyme was distinct from other previously reported GH3 enzymes in terms of amino acid sequence identity and enzymatic properties. GlyA_1_ from bovine ruminal fluid, which was the first reported GH3 enzyme with a permuted C-FnII-N domain topology, is a bifunctional enzyme with both *β*-glucosidase and *β*-xylosidase activities and lower activities toward other glycosides [11]. GlyA_1_ did not show any activity toward *β*-1,3 linkages, whereas our BrGH3A could hydrolyze both laminaribiose and laminaritriose. Other GH3 enzymes from the rumen metagenomes adopt the typical N-C-FnIII domain arrangement [4, 9, 10]. The kinetic parameters of these enzymes toward *β*-1,3-linked substrates were either very low [4] or not reported [9, 10]. In contrast, the previously reported GH3 enzymes with hydrolytic activities toward *β*-1,3-linked glucoside substrates were the two-domain *Hv*ExoI and *Hv*ExoII from germinated barley [31]. Both ExoI and ExoII are classified as *β*-D-glucan exohydrolases with a strong preference for laminaran polysaccharides. Their catalytic efficiency factors were 27- and 2-fold lower, respectively, toward laminaribose, and 250- and 5-fold lower, respectively, toward *p*NP-Glc. In contrast, our BrGH3A is a *β*-glucosidase with high activity toward laminaribiose and *p*NP-Glc but with little activity toward laminaripentaose. Therefore, the physiological functions of BrGH3A likely differ from those of other previously reported GH3 glycoside hydrolases.

A previous phylogenetic study demonstrated that GH3 enzymes with a permuted domain architecture are commonly present in Firmicutes and possibly also present in Actinobacteria and Archaea, especially those exposed to plant biomass in the digestive tracts of animals [11]. It has been proposed that domain rearrangement may offer novel enzymatic activities that are beneficial in such a unique ecological situation. Together with GlyA_1_ and *P*. *barengoltzii β*-glucosidase, BrGH3A may represent a member of a new clan with a permuted domain topology within the GH3 family. Due to its high sequence identity (98%), we proposed that BrGH3A belongs to *Spirochaetales* bacteria, which constitute >1% of the rumen microbiome and contribute to the digestion of hemicellulose and pectin polysaccharides [8, 32]. The lack of a signal peptide suggested that BrGH3A may act intracellularly in polysaccharide metabolism (termed the “selfish mechanism”), which is consistent with the absence of a secretory signal in >80% of carbohydrate-active enzymes predicted in metagenome-assembled rumen Spirochaetes genomes [8]. While *Spirochaetales* bacteria is not the major lignocellulose degrader among the ruminal microbiota, its role in carbohydrate metabolism could help relieve feedback inhibition of ruminal enzymes resulting from an accumulation of lignocellulolytic products. The kinetic properties of BrGH3A suggested its possible function as a GH3 *β*-glucosidase in the hydrolysis of laminarin-oligosaccharides released from the degradation of mixed-linkage glucans (containing both *β*-1,3- and *β*-1,4-linkages) in the cell wall of *Poales* grasses such as maize and rice, which typically make up animal feeds for cattle [8, 33]. Therefore, BrGH3A represents a novel exo-acting *β*-glucosidase from *Spirochaetales* bacteria that plays a role in the intracellular breakdown and utilization of degraded *β*-1,3-glucans.

## Conclusions

We identified a novel 2,445-bp GH3 *β*-glucosidase gene known as *BrGH3A* from the metagenome of the bovine rumen microbiota. After translation, BrGH3A is composed of 814 amino acid residues, with a predicted molecular weight of 91.7 kDa. Structural prediction demonstrated that BrGH3A has a permuted domain topology consisting of an N-terminal (*α*/*β*)_6_ sandwich domain, an FnIII domain, and a C-terminal (*β*/*α*)_8_ barrel-like domain. BrGH3A showed high catalytic efficiency toward laminaribiose and laminaritriose but low efficiency toward cellobiose. The amino acid sequence identity and kinetic properties suggest that BrGH3A is likely a novel *β*-glucosidase from *Spirochaetales* bacteria that is responsible for the breakdown of mixed-linkage glucans present in the cell wall of grass feeds. BrGH3A is yet another example of rich diversity in rumen hydrolytic enzymes and may represent a member of a new clan with a permuted domain topology within the GH3 family. Further study on its molecular structure in relation to the substrate specificity and domain architectures will improve our understanding of the biomass-degrading enzymes as well as their biocatalytic applications.

## Supporting information

S1 Fig Nucleotide and deduced amino acid sequences of the 1,084-bp PCR product obtained from the initial amplification of the bovine ruminal fluid metagenome with the degenerate CFN_for and CFN_rev primers. The nucleotide sequences corresponding to the degenerate CFN_for and CFN_rev primers at the 5’ and 3’ ends of the PCR product, respectively, are underlined. The amino acid sequence that showed 51% identity to the *β*-glucosidase-related glycosidase (accession number AFN84577.1) is highlighted in grey. The nucleotide sequences of the pGEM-T Easy vector are italicized.
(PDF)

S2 Fig Alignment of amino acid sequence deduced from the 1,084-bp PCR product and those of the uncultured bacterium glycosidase (accession number AFN84577.1) and a *Butyrivibrio* sp. GH3 protein (accession number WP_044930885.1).
(PDF)

S3 Fig Nucleotide and deduced amino acid sequences of BrGH3A identified from the bovine ruminal fluid metagenome. The nucleotide sequences corresponding to those of the degenerate primers are underlined. The PFGFGLSYT motif of the C-terminal domain and the GRNFEYYSEDP motif of the N-terminal domain are located at residues 273–281 and 625–635, respectively (grey highlights). The predicted acid/base catalyst and catalytic nucleophile are Glu148 and Asp739, respectively (yellow highlights).
(PDF)

S4 Fig Amino acid sequence alignment of BrGH3A with two other GH3 *β*-glucosidases with similarly permuted domain arrangement. 5WUG is from *P*. *barengoltzii* with 33% identity to BrGH3A [27], and 5K6L is GlyA_1_ from cow rumen metagenome with 32% identity to BrGH3A [11]. The conserved GRNFEYYSEDP and PFGFGLSYT motifs in the N- and C-domains of GH3 enzymes, respectively, are underlined. For BrGH3A, the (*α*/*β*)_6_ sandwich domain (residues Met1-Arg221, cyan), the FnIII domain (residues Ser282-Pro406, green), and the (*β*/*α*)_8_ barrel-like domain (residues Asp448-Val814, magenta) are indicated. The three loops (residues Thr472-Ala486, Ile521-Gln568, and Val741-Pro759) within the (*β*/*α*)_8_ barrel domain of BrGH3A are highlighted in grey. The predicted acid/base catalyst Glu148 and catalytic nucleophile Asp739 of BrGH3A are highlighted in yellow. The residues within the subsite –1 are marked with λ above the sequence.
(PDF)

S1 Table Nucleotide sequences of primers (5′ to 3′). (PDF)

S1 Raw images The original images of Figs 3 and 5. (PDF)

S1 Data Raw data. (XLSX)
