# Supplementary material for: Characterization of BrGH3A, a bovine rumen-derived glycoside hydrolase family 3 β-glucosidase with a permuted domain arrangement
Source: PLoS One. 2024 Jul 9;19(7):e0305817. doi: 10.1371/journal.pone.0305817 (PMC11233000; doi:10.1371/journal.pone.0305817)
Supplement: S1 Fig — The nucleotide sequences corresponding to the degenerate CFN_for and CFN_rev primers at the 5’ and 3’ ends of the PCR product, respectively, are underlined. The amino acid sequence that showed 51% identity to the β-glucosidase-related glycosidase (accession number AFN84577.1) is highlighted in grey. The nucleotide sequences of the pGEM-T Easy vector are italicized. (PDF) [file pone.0305817.s001.pdf]

ggggcagcatttacgtgacctatagaatactcaagctatgcatccaacgcgttgggagctc

G Q H L R D L - N T Q A M H P T R W E L

*tcccatatggtcgacctgcaggcggccgcgaattcactagtgatt*ccatttggttacggc

S H M V D L Q A A A N S L V I P F G Y G

ttgacatacacctcgttcaagaccgatgccagacctgtggagcttgaaaacgacacggta

L T Y T S F K T D A R P V E L E N D T V

aaagtagagatagacgtcacaaacaccggaaagcactccggcaaggagattgtccaggtc

K V E I D V T N T G K H S G K E I V Q V

tacgcaagctgccccggaggcaggctggacaagccatatcaggatccggcaggctttgca

Y A S C P G G R L D K P Y Q D P A G F A

aagacaaaggagctcaaaccgggcgagacccagactgtttcagtcagcttctgcatgaag

K T K E L K P G E T Q T V S V S F C M K

gaccttgcctcctacgacacggaatcctcctccttcatcctcgagaaaggcgactatgtc

D L A S Y D T E S S S F I L E K G D Y V

ataagaagcgggaactcaagtgccgccacagtgcccatcgcagtgatcagactggatgag

I R S G N S S A A T V P I A V I R L D E

gatgcaatcgtgctcaaggcaaaccctgctgcggcaaacctgatttcacggactggaag

D A I V L K A K P C C G K P D F T D W K

ccagacaatccctgcagggaggaaattccttcattcgttccggtcctgcagctcaaagcc

P D N P C R E E I P S F V P V L Q L K A

tcaaccatcgggacaaggagtgtggactacgattcccactaccccatcgatgatgaagtc

S T I G T R S V D Y D S H Y P I D D E V

agaaagctcaccgacagccagcttatctatgcgaacatagggaccttcaaggaaaacgca

R K L T D S Q L I Y A N I G T F K E N A

ggtcctctgagcgtcattggaagcgccagcgcacaggtggcgggagctgcgggccaggtc

G P L S V I G S A S A Q V A G A A G Q V

aacacaaagctcaacgatgtgggcttcaggacaatggtgctggcggacggtcctgccgga

N T K L N D V G F R T M V L A D G P A G

ctgagactcatccagcacttctacagggatggaaaaggcgcccacggactcggatcatca

L R L I Q H F Y R D G K G A H G L G S S

tcccattcgggcagtttcatggaatacctgcccaaggttctcaggttcttaatggatctt

S H S G S F M E Y L P K V L R F L M D L

ggaagaaggtccaaacctccccgaggcaaacaggaggaaagccagtactgcacggcaatc

G R R S K P P R G K Q E E S Q Y C T A I

cctatcggaacagccattgcccagagctggaacactgaattcgcccgcctttgcggagac

P I G T A I A Q S W N T E F A R L C G D

attgtcggaaccgagatggagatgtacggaatccagctctggctggcccctgccctcaac

I V G T E M E M Y G I Q L W L A P A L N

atccacaggtctatcctctgcggaagaaatttcgaatactattccgaag*aatcgaattcc*

I H R S I L C G R N F E Y Y S E E S N S

cgcggccgccatggcggccggagcatgcgactcccctgcct

R G R H G G R S M R L P C

S1 Fig. Nucleotide and deduced amino acid sequences of the 1,084-bp PCR product obtained from the initial amplification of the bovine ruminal fluid metagenome with the degenerate CFN_for and CFN_rev primers. The nucleotide sequences corresponding to the degenerate CFN_for and CFN_rev primers at the 5’ and 3’ ends of the PCR product, respectively, are underlined. The amino acid sequence that showed 51% identity to the *β*-glucosidase-related glycosidase (accession number AFN84577.1) is highlighted in grey. The nucleotide sequences of the pGEM-T Easy vector are italicized.
