# Supplementary material for: Characterization of BrGH3A, a bovine rumen-derived glycoside hydrolase family 3 β-glucosidase with a permuted domain arrangement
Source: PLoS One. 2024 Jul 9;19(7):e0305817. doi: 10.1371/journal.pone.0305817 (PMC11233000; doi:10.1371/journal.pone.0305817)
Supplement: S2 Fig — GH3 protein (accession number WP_044930885.1). (PDF) [file pone.0305817.s002.pdf]

```
PCR_product         ------------------------------------------------------------  0
AFN84577.1          MEMNTYEKEHSALMRRAGAECALFLKRGSGFPLDAPCEVALYGSGARNTLKGGTGSGDVN  60
WP_044930885.1      --MQQYEKDHLNLILDNAAECTVLLKANDAFPISKPCKIAAYGAGIRHTVMGGTGSGEVN  58


PCR_product         ------------------------------------------------------------  0
AFN84577.1          SRCFVTVEQALQGAGFTVTTKDWLTAYEEERGAARRKFIKQIKTEARKNHRNAIMEGMGA  120
WP_044930885.1      TRFSYTIEQGLEKEGFEITSKKWLDEYDAVRKAANKQFFKDLTKEAKAKHENPIMYTMGK  118


PCR_product         ------------------------------------------------------------  0
AFN84577.1          VMPEPEYDIPLNGSGHTAIYVLSRISGEGSDRSPVPGDILLSRTEVRDILALQNQYQKFM  180
WP_044930885.1      AMPEPFYQIPLDAKGDAAIYVVRRDSGEGNDRSVVEGDVKLARSEVRDILELNSKFKHFM  178


PCR_product         ------------------------------------------------------------  0
AFN84577.1          LVLNVGGVVDLSPVLNVENILLLSQLGVETGNVLADILLGKANPSGKLSATWSAWRDYPT  240
WP_044930885.1      LVLNVGGVVDLSPVMEVENILILSQLGVDCGKVLADIITGKQNPSGKLTTTWAAWEEYSK  238


PCR_product         --------------------------------PFGYGLTYTSFKTDARPVELENDTVKVE  28
AFN84577.1          IGAFGCFDDTPYTEGVYVGYRYFDSVGKKPLFPFGFGLSYTEFTVRPGTVTLDGETVTVT  300
WP_044930885.1      EGTFGDFNDNEYREGIYVGYRYFDTFGKKALFPFGYGLSYTTFEISAYKIVENADCVTLK  298
                                                    ***:**:** *      :  : : *.:

PCR_product         IDVTNTGKHSGKEIVQVYASCPGGRLDKPYQDPAGFAKTKELKPGETQTVSVSFCMKDLA  88
AFN84577.1          AAVTNTGSRAGREVVQIYVSSPAGKLDKPYQALAAFAKTRTLEPGETEEVTMNFKLRELA  360
WP_044930885.1      AKVKNTGLHEGKEVVQVYVSLPSGKLDKAYQELAGFAKTSLLKPGEEEQVEITFNLSDLA  358
                      *.*** : *:*:**:*.* *.*:*** **  *.****  *:*** : * :.* : :**

PCR_product         SYDTESSSFILEKGDYVIRSGNSSAATVPIAVIRLDEDAIVLKAKPCCGKPDFTDWKPDN  148
AFN84577.1          SYDEDMSAWVLERGDYILRVGSSSADTVPTAILRLENDAVTVQARSCFGKTGFTDWKPEC  420
WP_044930885.1      SYDEETASFILEKGDYIVRVGNSSASTKVVAAIELDETVTTLKVKNCLGDPGFEDIKAPY  418
                    *** : ::::**:***::* *.*** *    * :.*:: . .::.: * *. .* * *

PCR_product         PCR---------------EEIPSFVPVLQLKASTIGTRSVDYDSHYPIDDEVRKLTDSQL  193
AFN84577.1          PAV---------------AEAPDGLYVLQIPAGDIVTQTVAYDVTRPIDPAIDDLTDNEL  465
WP_044930885.1      WDNHKRNALEIGKVIFSNGKDSSGIQKLSISFKAISQETVSYKSDYEIDDVVKSLTDEEL  478
                                       :  . :  *.:    *  .:* *.    **  : .***.:*

PCR_product         IYANIGTFKENAGPLSVIGSASAQVAGAAGQVNTKLNDVGFRTMVLADGPAGLRLIQHFY  253
AFN84577.1          AYMSVGAFDPKAGVLSVIGNASQSVAGAAGETCGMLKDKGIPVIVMADGPAGLRLSRDYT  525
WP_044930885.1      CFLGIGGFNPNAKGLSVIGNAATHVAGAAGETTSELFLRGIKPLIMADGPAGLRLSKLYY  538
                     : .:* *. :*  *****.*:  ******:.   *   *:  :::********* : :

PCR_product         RDGKGAHGLGSS-SHSGSFMEYLPKVLRFLMDL-GRRSKPPRGKQEESQYCTAIPIGTAI  311
AFN84577.1          VDAKGVHPIGQ--TMPDGIIDFMPAPVRWFMNR--SGSRVKPGTDVRRQYCTAMPIGTAL  581
WP_044930885.1      EDEKGAHSADGAGMIPESMLEAMGPVVRFIATKVVGGKKAPKGAEIKEHYATRIPIGTAI  598
                     * **.*  .       .::: :   :*::       .:   * : . :*.* :*****:

PCR_product         AQSWNTEFARLCGDIVGTEMEMYGIQLWLAPALNIHRSILCGRNFEYYSE----------  361
AFN84577.1          AQSWDLELAEQCGDIVGDEMERFGVRLWLAPALNIQRDIRCGRNFEYYSEDPLLSGKLAA  641
WP_044930885.1      AQSFNTELAKAFGDMVGSEMEMMGIHLWLAPALNIHRSILCGRNFEYYSEDPLVSGLMAA  658
                    ***:: *:*.  **:** ***  *::*********:*.* **********

PCR_product         ------------------------------------------------------------  361
AFN84577.1          AVTRGVQKHPGRAVTIKHFAANNQETNRYSNNSQVSERALREIYLRGFGVCVTEGQPKAL  701
WP_044930885.1      AITEGVQNHRGCATTIKHYAANNAETNRYCNNSHVSERAMREIYLRGFGICVRKSQPHAL  718


PCR_product         ------------------------------------------------------------  361
AFN84577.1          MTSYNLINGVHTSEHRELIEDILRCEFGFDGVVMTDWIVSIGMQSKEAVHPAPNAGRIAA  761
WP_044930885.1      MTSYNLLNGTHTSEHRGLIEDILRCEFGFEGIVMTDWVMTVM-DSKKSIYRNAVSNEVAA  777


PCR_product         ------------------------------------------------------------  361
AFN84577.1          AGNDLTMPGCNDDYRAIMKALADGTLTREQLAINVSRIYRLAKALAED------------  809
WP_044930885.1      AGGDLFMPGGKKDYENVLKALRDGRLSREQLEINATRVVRMTEKLMNSCAEYADYNLELR  837


PCR_product         -- 361
AFN84577.1          -- 809
WP_044930885.1      IG 839
```

S2 Fig. Alignment of amino acid sequence deduced from the 1,084-bp PCR product and those of the uncultured bacterium glycosidase (accession number AFN84577.1) and a *Butyrivibrio* sp. GH3 protein (accession number WP_044930885.1).
