# Supplementary material for: Characterization of BrGH3A, a bovine rumen-derived glycoside hydrolase family 3 β-glucosidase with a permuted domain arrangement
Source: PLoS One. 2024 Jul 9;19(7):e0305817. doi: 10.1371/journal.pone.0305817 (PMC11233000; doi:10.1371/journal.pone.0305817)
Supplement: S3 Fig — The nucleotide sequences corresponding to those of the degenerate primers are underlined. The PFGFGLSYT motif of the C-terminal domain and the GRNFEYYSEDP motif of the N-terminal domain are located at residues 273–281 and 625–635, respectively (grey highlights). The predicted acid/base catalyst and catalytic nucleophile are Glu148 and Asp739, respectively (yellow highlights). (PDF) [file pone.0305817.s003.pdf]

```
atgcagctgcttgaccatgaaagaagacacatcggggcactgaggccctatcttgccgaa
 M  Q  L  L  D  H  E  R  R  H  I  G  A  L  R  P  Y  L  A  E
tgcacggttcttctgagaaaaaccgacgcattcccgctgaaagagccatgcagcatcgca
 C  T  V  L  L  R  K  T  D  A  F  P  L  K  E  P  C  S  I  A
cttcacggaaacggagcaaggcgcaccgtcaagggtggtacgggctccggagaggtcaac
 L  H  G  N  G  A  R  R  T  V  K  G  G  T  G  S  G  E  V  N
tcgaggtttttcgtcaacgtcgaggacggcctcgagcaggcgggattcaccgtcaccaca
 S  R  F  F  V  N  V  E  D  G  L  E  Q  A  G  F  T  V  T  T
aagaaatggctggatgcttacgattcaatccgcatcgaagcgaaaaaacacttcatggat
 K  K  W  L  D  A  Y  D  S  I  R  I  E  A  K  K  H  F  M  D
accgtcagggccgaggcaagggcacatcaccagatggccatcatgtttgccatgggaatg
 T  V  R  A  E  A  R  A  H  H  Q  M  A  I  M  F  A  M  G  M
gtcatgcccgagccagaatatgacctcgagctcgacctttcagcccaggctgccgtctat
 V  M  P  E  P  E  Y  D  L  E  L  D  L  S  A  Q  A  A  V  Y
gtcctttcccgagactccggagaaggcaacgacagaaggccggtcaagggcgatgttttt
 V  L  S  R  D  S  G  E  G  N  D  R  R  P  V  K  G  D  V  F
ctcacggattcggaaaaacgggacatccttgccctgaacaaggcatacaaaaagttcatg
 L  T  D  S  E  K  R  D  I  L  A  L  N  K  A  Y  K  K  F  M
cttgtcctcaatgtcggaggacccgtggacctgagaggactcgagagtgtgggaaacatt
 L  V  L  N  V  G  G  P  V  D  L  R  G  L  E  S  V  G  N  I
ctccttctttcccagctcggagtcgagacgggctgcgcacttgccgacatactgctcgga
 L  L  L  S  Q  L  G  V  E  T  G  C  A  L  A  D  I  L  L  G
cgggaaacccccttccggaaaactggcaaccacatggaccgcatgggacgactaccagagc
 R  E  T  P  S  G  K  L  A  T  T  W  T  A  W  D  D  Y  Q  S
atcggcactttcggagacaacgacgacacctattacaaagaaggcatctacgtcggatac
 I  G  T  F  G  D  N  D  D  T  Y  Y  K  E  G  I  Y  V  G  Y
aggtatttcaattccatagggaagcagaccatgttcccgttcggattcggcctttcctac
 R  Y  F  N  S  I  G  K  Q  T  M  F  P  F  G  F  G  L  S  Y
acctcgttcaagaccgatgccagacctgtggagcttgaaaacgacacggtaaaagtagag
 T  S  F  K  T  D  A  R  P  V  E  L  E  N  D  T  V  K  V  E
atagacgtcacaaacaccggaaagcactccggcaaggagattgtccaggtctacgcaagc
 I  D  V  T  N  T  G  K  H  S  G  K  E  I  V  Q  V  Y  A  S
tgccccggaggcaggctggacaagccatatcaggatctggcaggctttgcaaagacaaag
 C  P  G  G  R  L  D  K  P  Y  Q  D  L  A  G  F  A  K  T  K
gagctcaaaccgggcgagacccagactgtttcagtcagcttctgcatgaaggaccttgcc
 E  L  K  P  G  E  T  Q  T  V  S  V  S  F  C  M  K  D  L  A
tcctacgacacagaatcctcctccttcatcctcgagaaaggcgactatgtcataagaagc
 S  Y  D  T  E  S  S  S  F  I  L  E  K  G  D  Y  V  I  R  S
gggaactcaagtgccgccacagtgcccatcgcagtgatcagactggatgaggatgcaatc
 G  N  S  S  A  A  T  V  P  I  A  V  I  R  L  D  E  D  A  I
gtgctcaaggcaaaaccctgctgcggcaaacctgatttcacggactggaagccagacaat
 V  L  K  A  K  P  C  C  G  K  P  D  F  T  D  W  K  P  D  N
ccctgcagggaggagattccatcattcgttccggtcctgcagctcaaagcctcaaccatc
 P  C  R  E  E  I  P  S  F  V  P  V  L  Q  L  K  A  S  T  I
gggacaaggagcgtggactacgattctcactaccccatcgatgatgaagtcagaaagctc
 G  T  R  S  V  D  Y  D  S  H  Y  P  I  D  D  E  V  R  K  L
accgacagccagcttatctatgcgaacatagggaccttcaaggaaaacgcaggtccactg
 T  D  S  Q  L  I  Y  A  N  I  G  T  F  K  E  N  A  G  P  L
agcgtcattggaagcgccagcgcacaggtggcgggagctgcaggccaggtcaacacaaag
 S  V  I  G  S  A  S  A  Q  V  A  G  A  A  G  Q  V  N  T  K
ctcaatgatgtgggcttcaggacaatggtgctggcggacggtcctgccggactgagactc
 L  N  D  V  G  F  R  T  M  V  L  A  D  G  P  A  G  L  R  L
atccagcacttctacagggacggaaaaggcgcccacggactcggatcatcatcccattcg
 I  Q  H  F  Y  R  D  G  K  G  A  H  G  L  G  S  S  S  H  S
ggcagcttcatggaatacctgcccaaggttctaagattcctcatggaccttggaagaagg
 G  S  F  M  E  Y  L  P  K  V  L  R  F  L  M  D  L  G  R  R
tccaaaccgcccagaggcaaacaggaggaaagccagtactgcacggcaattcccatcgga
 S  K  P  P  R  G  K  Q  E  E  S  Q  Y  C  T  A  I  P  I  G
actgccatagctcagagctggaacactgaattcgcccgcctttgcggagacattgtcgga
 T  A  I  A  Q  S  W  N  T  E  F  A  R  L  C  G  D  I  V  G
accgagatggagatgtacggtgtccagctctggctggcccctgccctcaacatccacagg
 T  E  M  E  M  Y  G  V  Q  L  W  L  A  P  A  L  N  I  H  R
tctatcctctgcggaaggaacttcgaatactattcggaggacccgcttgtcagcggcatg
 S  I  L  C  G  R  N  F  E  Y  Y  S  E  D  P  L  V  S  G  M
atggcggcatccatcacgatcggagtccagaaccacaaaggctgcggaacaaccatcaag
 M  A  A  S  I  T  I  G  V  Q  N  H  K  G  C  G  T  T  I  K
cattatgccaccaacaaccaggagacaaaccgttacggaaacagcagcaacgtctccgaa
```

```
 H  Y  A  T  N  N  Q  E  T  N  R  Y  G  N  S  S  N  V  S  E 
cgtgctctcagggaaatctatctgaaaggcttcggcctctgcgtcaggctgtctcagccg
 R  A  L  R  E  I  Y  L  K  G  F  G  L  C  V  R  L  S  Q  P 
aagtctgtcatgacatcctacaaccttctcaacggaaagcacacggccgaaagcagggat
 K  S  V  M  T  S  Y  N  L  L  N  G  K  H  T  A  E  S  R  D 
ctgatcgagagcatactcagatgcgagttcggcttcaagggaattgtaatgaccgactgg
 L  I  E  S  I  L  R  C  E  F  G  F  K  G  I  V  M  T  D  W 
gtcgtatcggacggaataggaaacaatccgaaggacatccatccaaaggtgaaaccccag
 V  V  S  D  G  I  G  N  N  P  K  D  I  H  P  K  V  K  P  Q 
ctgacggcagcagccggaagcgacctgttcatgccgggatgcaagaaggactacaacaac
 L  T  A  A  A  G  S  D  L  F  M  P  G  C  K  K  D  Y  N  N 
atgatggccggcctcgcagacggatcggtgaccagagagcagcttcagataaacgccacc
 M  M  A  G  L  A  D  G  S  V  T  R  E  Q  L  Q  I  N  A  T 
cgggtctaccggatggcgaaagaactcagtgatggaaaagtctga
 R  V  Y  R  M  A  K  E  L  S  D  G  K  V  - 
```

S3 Fig. Nucleotide and deduced amino acid sequences of BrGH3A identified from the bovine ruminal fluid metagenome. The nucleotide sequences corresponding to those of the degenerate primers are underlined. The PFGFGLSYT motif of the C-terminal domain and the GRNFEYYSEDP motif of the N-terminal domain are located at residues 273-281 and 625-635, respectively (grey highlights). The predicted acid/base catalyst and catalytic nucleophile are Glu148 and Asp739, respectively (yellow highlights).
