# Supplementary material for: Characterization of BrGH3A, a bovine rumen-derived glycoside hydrolase family 3 β-glucosidase with a permuted domain arrangement
Source: PLoS One. 2024 Jul 9;19(7):e0305817. doi: 10.1371/journal.pone.0305817 (PMC11233000; doi:10.1371/journal.pone.0305817)
Supplement: S4 Fig — 5WUG is from P. barengoltzii with 33% identity to BrGH3A [27], and 5K6L is GlyA1 from cow rumen metagenome with 32% identity to BrGH3A [11]. The conserved GRNFEYYSEDP and PFGFGLSYT motifs in the N- and C-domains of GH3 enzymes, respectively, are underlined. For BrGH3A, the (α/β)6 sandwich domain (residues Met1-Arg221, cyan), the FnIII domain (residues Ser282-Pro406, green), and the (β/α)8 barrel-like domain (residues Asp448-Val814, magenta) are indicated. The three loops (residues Thr472-Ala486, Ile521-Gln568, and Val741-Pro759) within the (β/α)8 barrel domain of BrGH3A are highlighted in grey. The predicted acid/base catalyst Glu148 and catalytic nucleophile Asp739 of BrGH3A are highlighted in yellow. The residues within the subsite –1 are marked with λ above the sequence. (PDF) [file pone.0305817.s004.pdf]

```
BrGH3A        ----------------------------MQLLDHERRHIGALRPYLAECTVLLRKTDAFPL   33
5WUG          MGSSHHHHHHSSGLVPRGSHMASMSKSMLGVPLEGFAEYSRIAAAEGGVLLKNENAMLPI   60
5K6L          -----------------------MNIEKVILDWNEYIEAARSVVSEGC-VLLENNGTLPL   36
                                             :  :     :         :* .:.  :*:

BrGH3A        KEPCSIALHGNGARRTVKGGTGSG-EVNSRFFVNVEDGLEQAGFTVTTKKWLDAYDSIRI   92
5WUG          RAHEIVSVFGRCQIDYYRSGTGSGGAVNVPYVVNILDGLRANPRIQVNEQLAKQYEQWIA   120
5K6L          EKGAVVSIFGRIQTHYYKSGTGSGGMVNVTHVVGVPEGLKLSEHVTVNEELENIYKEWEE   96
              .    :::.*.       :.*****  **   ..*.: :**.       ..::  . *..

                                                                       ●
BrGH3A        EAKKHFMDT---VRAEARAHHQMAIMFAMGMVMPEPEYDLELDLSAQAAVYVLSRDSGEG   149
5WUG          E--NPFDNGGGGWAAEPWCQKEMPL---------TDEIVAQAKQASSKAIVIIGRTAGED   169
5K6L          E--NPFDEG-LGWGTEPWSQPEMEL---------TDEIVSNASAKSDVAIVIIGRTAGED   144
              *  : * :       :*  .: :* :           *    : .  :. *: ::.* :**.

BrGH3A        NDRRPVKGDVFLTDSEKRDILALNKAYKKFMLVLNVGGPVDLRGLES------VGNILLL   203
5WUG          KDNADTEGSYRLTEQERLNLETVTRHFDQVAVLMNVANVIDMSWINDPVHQGRIRAVMFV   229
5K6L          KDFSDVAGAYKLSETEEDMLRRVRKHFDKMVVLLNVGSLMDLNVISEI----NPDALMVI   200
              :*    . *    *:: *.   :   : : :.:. :::**.. :*:   :..         ::.:

BrGH3A        SQLGVETGCALADILLGRETPSGKLATTWT-AWDDYQSIGTFGDNDDTYYKEGIYVGYRY   262
5WUG          WQGGMIGGHAVADLLSGDVTPSGKLPDTIAHHIEDYPSTANFGSEERNLYEEDIYVGYRY   289
5K6L          WQGGMIGGLGTADVLTGKVNPSGKLTDTIAYEINDYPSTENFGDPVRDYYAEDIYVGYRY   260
               * *:   *  . **:* *  .*****  * :    :** *   .**.       * *.*******

BrGH3A        FNSIGK-QTMFPFGFGLSYTSFKTDARPVELEND----TVKVEIDVTNT-GKHSGKEIVQ   316
5WUG          FETFCPDKVLFPFGYGLSYTSFAWKVQGVKLEGAGTDAQLEVQVEVTNTGSEFSGKEVIQ   349
5K6L          FETFEKSKVRYPFGYGISYTEFEHTVGEFTADINSR--TFTASCTVKNT-GSVAGKDVAQ   317
              *:::    :. :***:*:***.*     .   .   :          .  ..   *.**  ..  :**::  *

BrGH3A        VYASCPGGRLDKPYQDLAGFAKTKELKPGETQTVSVSFCMKDLASYDT-----ESSSFIL   371
5WUG          LYYEAPQGVLGKPARALGAFAKTKLLQPGESDVLTLQLPVRRMASYDDGGYTGHKSCYVL   409
5K6L          FYVSAPQGKLGKPEKVLVAFKKTGILNPGKEEKITVTVPFDRFASFDDTGVTGAESCFVL   377
              .*  ..* * *.** :  *  .* **  *:**: : ::: .  .   :**:*         .*.::*

BrGH3A        EKGDYVIRSGNSSAATVPIAV-----IRLDEDAIVLKAK-PCCGKPDFTDWKPDNP----   421
5WUG          EAGDYEFHVGNSIRNTERVTVDGKAAYQLAELMVVEQLEEAAAPTQRFSRLKPGRRKPDG   469
5K6L          EAGEYTVYEGKNVRESYK-----EGSFTLEENIVTEKLSKALAPMESFKRMKASE-NSDG   431
              *  *:*  .   *:.    :                 * *   :. : .    .      *.    *  ..

BrGH3A        ----CREEIPSFVPVLQLKASTIGTRSVDY----DSHYPIDD----------EVRKLTDS   463
5WUG          TYEIVREEVPQRT--ISLKERIERRLPEAYPQTGNRGIKLKDVQAGKASLEEFVAQLSDE   527
5K6L          TLSVKYEDVPVSD--VDEKKRRLDNMPVEIPQDFTARYSLKDVLSGSVDMEKFIARLSDD   489
                     *::*      :.  *                          :.*            :  :*:*.

                                                                ●
BrGH3A        QLIYANIGTFKENAGPLSVIGSASAQVAGAAGQVNTKLNDVGFRTMVLADGPAGLRLIQH   523
5WUG          DLAT-----IVRGEGMSS--PKVTPGTASAFGGVGENLLEYGIPVACTADGPSGIRM---   577
5K6L          DLAC-----IVRGEGMGS--SLVTAGTAAAFGGVSEYLRKMDIPAVCCDDGPSGMRL---   539
              :*         :  ..  *   *      .:   .*.*  *  *.    *   .  .:  .       ***:*:*:

BrGH3A        FYRDGKGAHGLGSSSHSGSFMEYLPKVLRFLMDLGRRSKPPRGKQEESQYCTAIPIGTAI   583
5WUG          ---------------------------------------------DSGLKATQLPIGTLL   592
5K6L          ---------------------------------------------DSGATAFSMPNGTML   554
                                                           :..   .   :*  ** :

                                                          ●
BrGH3A        AQSWNTEFARLCGDIVGTEMEMYGVQLWLAPALNIHRSILCGRNFEYYSEDPLVSGMMAA   643
5WUG          ASSWDVDLVESLYVLEGKELLQNEIDTLLGPGINIHRHPLNGRNFEYFSEDPYLTGCFAS   652
5K6L          ASTFNPDVIERMYGFTSLEMIYNKVECLLGPGMNIHRNPLNGRNFEYFSEDPYLNGTIAS   614
              *.::: :. .      :  .  *:      ::   *.*.:****   *  ******:****   :.*  :*:

                              ●●         ●
BrGH3A        SITIGVQNHKGCGTTIKHYATNNQETNRYGNSSNVSERALREIYLKGFGLCVRLSQPKSV   703
5WUG          AVTRGIKK-GGSSATVKHFAGNNQEKARSKVDAVVSERALREIYLKGFEMAVKEGEATSI   711
5K6L          AMLKGLHK-YGSDGVAKHFCCNNQELGRQACDSVVSQRALREIYLKGFEIAVKEGGCKAF   673
              ::   *:::    *..  .  **:.  ****    *       .:  **:***********  :.*:  .    .:.

                ●                          ●●
BrGH3A        MTSYNLLNGKHTAESRDLIESILRCEFGFKGIVMTDWVVSD--GIGNNPKDIHPKVKPQL   761
5WUG          MTSYNPVNGHWAASNYDLNTTILRNEWGYQGIVMTDWWAVMNDCVEGGPADLKN---TSF   768
5K6L          MTTYAQVNGMWTAGNYDLNTRILRDEWGFKGIVMTDWWAQVNDR--GGEPTKNN---TAA   728
              **:*   :**   :*  .  **     *** *:*::*******  .           ..      :

BrGH3A        TAAAGSDLFMPGCK------KDYNNMMAGLADGSVTREQLQINATRVYRMAKELSDGKV-   814
5WUG          MVRAQNDLYMVVNNDGAEINSLGDNTLEALANGTLTVGELQRCAMNICRFLLNAPALARE   828
5K6L          MVRAQNDLYMVTAN--AAMNSANDNTLSQLSEGKLNRAELQRCAMNICEYAMNTMAMKRL   786
               .  *  .**:*    :         .   :*  :    *::*.:.    :**    *  .:  .    :

BrGH3A        ------------------------------------------------------------   814
5WUG          PKPVHEVRLIQAAQG--DLPIASAGVNVYTLSRSQSAKVLA-------NAETAVVKVQEA   879
5K6L          CRNDIKVEIAGRVIEEDAFDIENAE---YLVLKGNITVSLKNKESKAGTNYYIPLDIQDL   843
```

```
BrGH3A      ------------------------------------------------------------   814
5WUG        GVYTVTAHIRYEAMNLSQSACNLLLNGELLTTVQTNGTLGRWVTQKQLRIELTEGDYELK   939
5K6L        GMYDISVTASSMLGEVAQLPCTLYYTGVPFLTYTFNGSGGKDVTITK-SMDFHNRMAVIR   902

BrGH3A      -------------------      814
5WUG        FDYIKPGLEIEWIEFI---      955
5K6L        LNVAKNGLNLDRIEFKKQQ      921
```

S4 Fig. Amino acid sequence alignment of BrGH3A with two other GH3 *β*-glucosidases with similarly permuted domain arrangement. 5WUG is from *P. barengoltzii* with 33% identity to BrGH3A [27], and 5K6L is $GlyA_1$ from cow rumen metagenome with 32% identity to BrGH3A [11]. The conserved GRNFEYYSEDP and PFGFGLSYT motifs in the N- and C-domains of GH3 enzymes, respectively, are underlined. For BrGH3A, the $(\alpha/\beta)_6$ sandwich domain (residues Met1-Arg221, cyan), the FnIII domain (residues Ser282-Pro406, green), and the $(\beta/\alpha)_8$ barrel-like domain (residues Asp448-Val814, magenta) are indicated. The three loops (residues Thr472-Ala486, Ile521-Gln568, and Val741-Pro759) within the $(\beta/\alpha)_8$ barrel domain of BrGH3A are highlighted in grey. The predicted acid/base catalyst Glu148 and catalytic nucleophile Asp739 of BrGH3A are highlighted in yellow. The residues within the subsite –1 are marked with ● above the sequence.
