# Supplementary material for: Characterization of BrGH3A, a bovine rumen-derived glycoside hydrolase family 3 β-glucosidase with a permuted domain arrangement
Source: PLoS One. 2024 Jul 9;19(7):e0305817. doi: 10.1371/journal.pone.0305817 (PMC11233000; doi:10.1371/journal.pone.0305817)
Supplement: S1 Table — (PDF) [file pone.0305817.s005.pdf]

S1 Table. Nucleotide sequences of primers (5′ to 3′)

Purpose	Primer	Nucleotide sequences
Initial amplification	NCF_for	GGH MGN AAY YWY GAR TAY TWY HSN GAR GAY CC
	NCF_rev	DGT RTA DSW MAD HCC RWR NCC RAA BGG
	CFN_for	CCN TTY GGH TWY GGV YTB WSM TAY AC
	CFN_rev	CYT CNG ARW ART AYT CRA ART TNC KKC C
Genome walking	GSP1_for	GAA CCG AGA TGG AGA TGT ACG GAA TCC
	GSP2_for	GGT CTA TCC TCT GCG GAA GAA ATT TCG
	GSP1_rev	TAC CGT GTC GTT TTC AAG CTC CAC AGG
	GSP2_rev	GGT GTA TGT CAA GCC GTA ACC AAA TGG
Cloning of *BrGH3A*	BrGH3A_for	CAT ATG CAG CTG CTT GAC CAT GAA AGA A[1]
	BrGH3A_rev	GGA TCC TCA GAC TTT TCC ATC ACT GAG TT[2]

Note: [1] The *Nde*I cutting site is underlined.

[2] The *Bam*HI cutting site is underlined.
