# Supplementary material for: Characterization of BrGH3A, a bovine rumen-derived glycoside hydrolase family 3 β-glucosidase with a permuted domain arrangement
Source: PLoS One. 2024 Jul 9;19(7):e0305817. doi: 10.1371/journal.pone.0305817 (PMC11233000; doi:10.1371/journal.pone.0305817)
Supplement: S1 Raw images — (PDF) [file pone.0305817.s006.pdf]

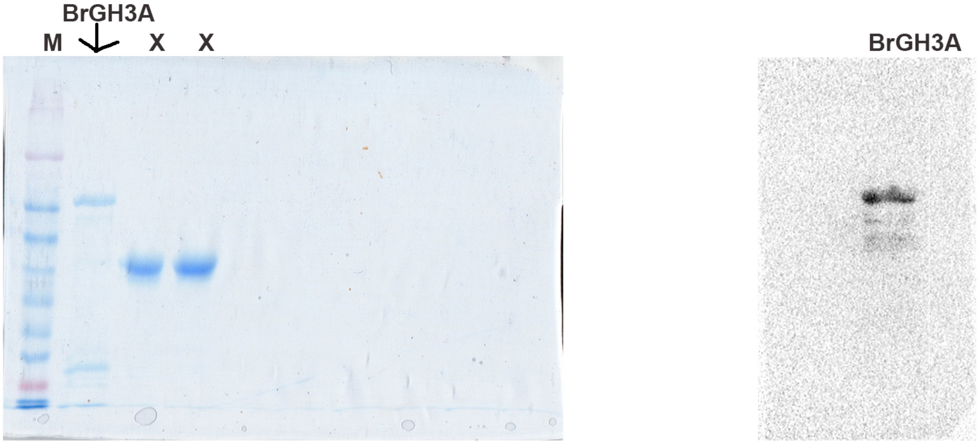


Fig 3. 10% SDS-PAGE analysis (left) and Western blot analysis (right) of the purified BrGH3A. Lane M is the protein size marker. Note that only the first 2 lanes of the gel were blotted.

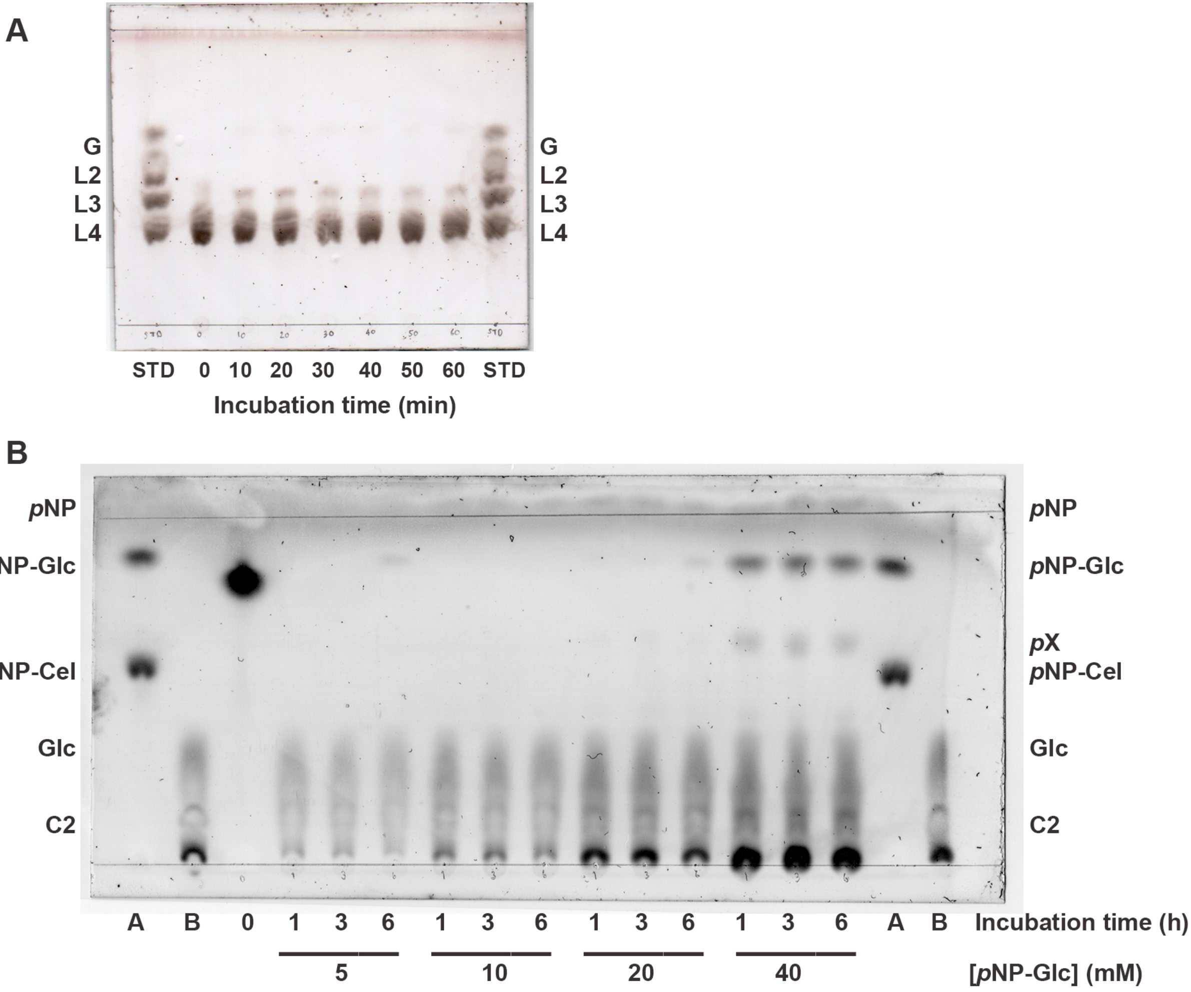


**Fig 5. Time-course reactions of BrGH3A with laminaritetraose (A) and *p*NP-Glc (B).**
